# The structure-sensitivity of memory access: evidence from Mandarin Chinese

**DOI:** 10.3389/fpsyg.2014.01025

**Published:** 2014-09-24

**Authors:** Brian Dillon, Wing-Yee Chow, Matthew Wagers, Taomei Guo, Fengqin Liu, Colin Phillips

**Affiliations:** ^1^Department of Linguistics, University of MassachusettsAmherst, MA, USA; ^2^Basque Center on Cognition, Brain and LanguageDonostia – San Sebastián, Spain; ^3^Department of Linguistics, University of California Santa CruzSanta Cruz, CA, USA; ^4^State Key Laboratory of Cognitive Neuroscience and Learning, Center for Collaboration and Innovation in Brain and Learning Sciences, Beijing Normal UniversityBeijing, China; ^5^State Key Laboratory of Cognitive Neuroscience and Learning, Beijing Normal UniversityBeijing, China; ^6^Department of Linguistics, Maryland Language Science Center, University of MarylandCollege Park, MD, USA

**Keywords:** working memory, reflexive processing, speed-accuracy trade-off, Mandarin Chinese, sentence processing

## Abstract

The present study examined the processing of the Mandarin Chinese long-distance reflexive *ziji* to evaluate the role that syntactic structure plays in the memory retrieval operations that support sentence comprehension. Using the multiple-response speed-accuracy tradeoff (MR-SAT) paradigm, we measured the speed with which comprehenders retrieve an antecedent for *ziji*. Our experimental materials contrasted sentences where *ziji*'s antecedent was in the local clause with sentences where *ziji*'s antecedent was in a distant clause. Time course results from MR-SAT suggest that *ziji* dependencies with syntactically distant antecedents are slower to process than syntactically local dependencies. To aid in interpreting the SAT data, we present a formal model of the antecedent retrieval process, and derive quantitative predictions about the time course of antecedent retrieval. The modeling results support the *Local Search hypothesis*: during syntactic retrieval, comprehenders initially limit memory search to the local syntactic domain. We argue that Local Search hypothesis has important implications for theories of locality effects in sentence comprehension. In particular, our results suggest that not all locality effects may be reduced to the effects of temporal decay and retrieval interference.

## Introduction

One fundamental question for models of sentence comprehension is the question of how comprehenders are able to construct long-distance linguistic dependencies reliably and rapidly in comprehension. Long-distance dependencies occur whenever two non-adjacent elements in a sentence must be syntactically and/or semantically integrated with each other. For example, in a sentence like “*William took a terrible yet interesting photo of himself*,” the relationship between the reflexive anaphor *himself* and its antecedent *William* is constructed across multiple intervening words. Recent models of sentence comprehension have advanced the hypothesis that this sort of syntactic dependency formation minimally requires the use of memory retrieval mechanisms to access temporally distant syntactic encodings. On this view, to interpret the reflexive in the sentence above, comprehenders must retrieve a representation of the antecedent from memory. Moreover, it has been argued that the memory retrieval mechanisms that underlie sentence comprehension share a number of key features with domain-general retrieval mechanisms (McElree, 2000; McElree et al., 2003; Van Dyke and Lewis, 2003; Lewis and Vasishth, 2005; Lewis et al., 2006). This view receives support from mounting evidence that comprehenders rely on a cue-based, direct access retrieval mechanism during syntactic comprehension. Cue-based retrieval mechanisms allow direct access to syntactic encodings by matching retrieval cues to the features of all item representations in memory in a parallel fashion. Items whose features provide a close match to the retrieval cues are then retrieved for further processing (for a discussion of implementations of this idea, see Clark and Gronlund, 1996).

Despite the growing evidence in favor of cue-based retrieval in sentence comprehension, these models still face a number of difficult theoretical questions. One central question concerns the nature of the retrieval cues used to retrieve syntactic dependents during processing (Van Dyke and McElree, 2011; Dillon et al., 2013; Kush, 2013). Existing evidence suggests that comprehenders use both semantic and syntactic cues to guide retrieval (Van Dyke and McElree, 2006, 2011; Van Dyke, 2007). Furthermore, there is some evidence that syntactic cues may be given priority over semantic or morphological cues, although this may depend on the kind of dependency being formed (Van Dyke and McElree, 2011; Dillon et al., 2013). However, very little is known about the nature of the syntactic cues that guide retrieval operations. In the present paper, we address this question by asking whether syntactic cues refer only to the attributes of individual syntactic encodings, such as their case or thematic role (*item information*), or whether syntactic cues distinguish constituents based on their hierarchical or linear distance from the retrieval site (*position information*). We hypothesize that the cues that guide memory retrieval during parsing do include positional syntactic information, and furthermore, that comprehenders use positional information as retrieval cues to prioritize retrieval of constituents within the local syntactic domain (what we will refer to as the *Local Search hypothesis*).

The goal of the present paper is to evaluate the Local Search hypothesis by examining the speed with which comprehenders process reflexive dependencies in Mandarin Chinese, where reflexive anaphors may be bound either inside or outside of their local clause. We then develop a formal model of a local search retrieval process, and derive quantitative predictions about the processing time necessary to recover *ziji*'s antecedent if the parser assumes a local search strategy. To preview our conclusion, the results of our investigation support the main predictions of the Local Search hypothesis. We argue that the Local Search hypothesis offers important insight into a widely-observed preference for local dependencies over distant dependencies in sentence comprehension (Kimball, 1973; Hawkins, 1994; Gibson, 1998; Bartek et al., 2011; *a*.*o*.). In particular, the results presented here support theories that attribute these *locality effects* to a substantive bias to search syntactically local domains at retrieval, rather than theories that attribute locality effects entirely to effects of decay or interference of items in working memory (MacDonald et al., 1994; Lewis and Vasishth, 2005; Lewis et al., 2006).

### Cue-based retrieval in sentence processing

There are two main sources of empirical evidence that implicate the use of a cue-based retrieval mechanism in parsing. The first source of evidence comes from studies of interference effects in online processing. In a cue-based, direct access memory architecture, the process of matching the retrieval cues against all memory encodings allows rapid access to task-relevant encodings, but is susceptible to interference effects. If there is a good match between the retrieval cues and more than one item in memory, then access to the target memory can be impeded. Similarity-based interference effects have been widely documented in studies of sentence comprehension (Gordon et al., 2001, 2004; Van Dyke and Lewis, 2003; Drenhaus et al., 2005; Lewis and Vasishth, 2005; Lewis et al., 2006; Van Dyke and McElree, 2006, 2011; Van Dyke, 2007). In addition, cue-based retrieval architectures naturally account for the phenomenon of *illusory licensing*. Illusory licensing occurs when comprehenders appear to use a grammatically unavailable constituent to license a syntactically dependent element (negative polarity item licensing: Vasishth et al., 2008; Xiang et al., 2009; subject-verb agreement: Wagers et al., 2009; Dillon et al., 2013). In a cue-based retrieval architecture, this arises because a syntactically illicit constituent may be misretrieved during the search for a licensor, which in turn leads to illusory licensing of the dependent element that triggered the retrieval.

A second important line of evidence in favor of cue-based retrieval mechanisms comes from studies of the time course of memory access. In a cue-based, direct access memory architecture, only items that match the retrieval cues are contacted at retrieval, and so retrieval times are predicted to be constant over search sets of different sizes. This prediction has been supported by speed-accuracy tradeoff studies (SAT) of item recognition. In an SAT study, participants are trained to respond at a number of varying response deadlines. This allows the experimenter to derive an SAT function that tracks behavioral accuracy as a function of time. This function measures the complete time course of information processing. Importantly, SAT permits the experimenter to make separate measurements of processing speed and processing accuracy, two aspects of information processing that are confounded in simple reaction time (RT) paradigms (Wickelgren, 1977). SAT studies of recognition judgments in list memory tasks have provided support for direct access models of recognition memory by showing that the number of elements in the list does not affect processing speed (McElree and Dosher, 1989). This finding is not consistent with search models, which retrieve representations based on their location in memory. Search may proceed either in serial or parallel, but it crucially involves performing explicit comparison processes over a positionally defined search set (see Townsend and Ashby, 1983). For this reason, search models predict that memory access times should grow either as a function of the size of the search space, or as a function of the position of the target in a serial or ordered search. This prediction reflects the fact that the more sampling operations are necessary to recover the intended target, the longer time should be required for retrieval. In contrast to item recognition, the retrieval of explicit order information does appear to recruit this sort of iterative search process (McElree and Dosher, 1993; Gronlund et al., 1997).

In language comprehension, research using the SAT technique has demonstrated that memory access time does not grow with the size of the search space (McElree, 2000; McElree et al., 2003; Foraker and McElree, 2007; Martin and McElree, 2008, 2009). For instance, McElree et al. (2003) examined the processing of object cleft-constructions as in (1), in which the clefted object is separated from the associated verb by 1, 2, or 3 clauses:

- It was the scandal that the celebrity relished.
- It was the scandal that model believed that the celebrity relished.
- It was the scandal that the model believed that the journalist reported that the celebrity relished.    (1)

McElree and colleagues used the SAT paradigm to measure comprehension accuracy at various time points from the offset of the final verb in (1), manipulating the hierarchical (and linear) distance between the filler (*the scandal*) and the verb that hosts its gap (*relished*). The results suggested that the length manipulation impacted how accurately comprehenders were able to retrieve the *wh*-filler, as reflected in their asymptotic accuracy rates on a plausibility detection task, but that it did not impact the speed of this retrieval. McElree et al. argued that these results favor a cue-based direct access retrieval mechanism over search-based retrieval mechanisms, on the assumption that their length manipulation increased the size of the search set for the critical retrieval of the *wh*-filler. Similar results were observed for the comprehension of verb phrase ellipsis (Martin and McElree, 2008, 2009), sluicing (Martin and McElree, 2011), and pronominal reference (Foraker and McElree, 2007).

Although previous SAT studies have consistently found that the structural distance between two elements in a dependency does not impact the speed of forming the dependency, the technique has been shown to have the power to detect other sorts of processing slowdowns that occur during sentence comprehension. For example, SAT studies have shown that processing slowdowns obtain in cases of syntactic reanalysis (McElree et al., 2003; Bornkessel et al., 2004), cases of potential lexical ambiguity (Foraker and McElree, 2007), and configurations that require multiple retrieval operations (McElree et al., 2003). Lastly, although length *per se* has not been shown to modulate processing speed, in certain cases the type of intervening material has been shown to contribute to slowed processing. McElree et al. (2003) also reported that the time necessary to process a subject-verb dependency is slowed by an intervening relative clause, but not an intervening prepositional phrase (see also Wagers and McElree, 2009).

### Locality effects in a cue-based architecture

The adoption of a cue-based, direct access architecture for syntactic processing requires a reexamination of existing theories of locality effects in sentence processing. It is widely observed that local syntactic dependencies are easier to process or, in cases of ambiguity, preferred over longer syntactic dependencies (Kimball, 1973; Frazier, 1978; Just and Carpenter, 1992; Hawkins, 1994; Gibson, 1998; Lewis and Vasishth, 2005; Lewis et al., 2006; Bartek et al., 2011; *inter alia*). Because cue-based, direct access mechanisms do not need to execute a serial search of a parse to retrieve a syntactic dependent or a pronominal antecedent, locality effects cannot emerge as a property of the access mechanism without making further assumptions. Instead, the advantage for local dependencies reflects two factors: time-based decay and interference processes (Van Dyke and Lewis, 2003; Lewis and Vasishth, 2005; Lewis et al., 2006; Bartek et al., 2011; see also Frazier and Clifton, 1998). Decay and interference both serve to degrade the availability of more distant syntactic constituents, thus making processing of longer dependencies more difficult. Decay does so by affecting the activation of constituents: more local constituents will have higher activation values by virtue of being accessed more recently. The effect of interference is more indirect. When dependencies are longer, then there are likely to be more constituent encodings in memory. When there are more items in memory, the degree of similarity-based interference is likely to be greater.

These explanations for locality effects in sentence processing stand in contrast to the explanation offered by accounts that attribute locality effects to a parsing strategy that preferentially attaches incoming constituents within a local syntactic domain (Kimball, 1973; Frazier, 1978; Berwick and Weinberg, 1984; Frazier and Clifton, 1989, 1996; Gibson et al., 1996; Gibson, 1998; Sturt et al., 1999, 2000). Although these accounts vary widely in their details, they might all be called *search-based* accounts of syntactic retrieval. The core claims of search-based accounts include (i) the parser distinguishes local vs. distant syntactic domains through positional syntactic information, and (ii) the search for a potential syntactic dependent proceeds by first searching within some local syntactic domain, the size of which may vary across theories.

Although it may appear that the claims of these search-based accounts are incompatible with the locality account advanced by cue-based parsing models, this is not so. The core claims of search-based accounts may in fact be integrated with cue-based retrieval models if we suppose that positional syntactic information is available to guide retrieval operations, and that at retrieval the parser uses this positional information to limit retrieval to a local syntactic domain. We call this the *Local Search* hypothesis:

*Local Search hypothesis*: The parser uses positional syntactic information during the retrieval of syntactic dependents, and positional cues serve to restrict retrieval to constituents in some local syntactic domain.    (2)

According to the Local Search hypothesis, locality effects in sentence processing reflect in part a parsing strategy that prioritizes the retrieval of syntactically local constituents. In other words, the Local Search hypothesis claims that locality effects in sentence processing do not merely emerge from effects of decay and interference, but instead they reflect a strategy for the retrieval of syntactic dependents.

Within existing cue-based parsing models, it is not generally assumed that positional syntactic information is available to guide retrieval. Lewis and Vasishth (2005) propose that positional syntactic information, either hierarchical or linear, plays no role in the memory retrieval operations that guide attachment operations (cf. the *no serial order* hypothesis; see also Lewis et al., 2006). For example, while the parser may be able to use item information such as case to identify the subject of a sentence, it cannot use positional syntactic information to distinguish the encoding of the local subject from a more distant subject. On this account, the inability to distinguish distant and local subjects on the basis of their syntactic position provides an explanation of the well-known center embedding difficulty in terms of retrieval difficulty: when the parser needs to retrieve a subject for an embedded verb, there are too many similar subject encodings in memory to permit the parser to retrieve the grammatically appropriate subject, and positional information cannot be recruited to help with this process (Lewis and Vasishth, 2005; Lewis et al., 2006). In contrast to the Local Search hypothesis, these models claim that locality effects in sentence processing are entirely reducible to the effects of interference and temporal decay.

This claim is compatible with the SAT results reviewed above, which suggest that neither the linear nor structural position of the retrieval target directly impacts retrieval times. It is tempting to conclude from this that positional syntactic information is not used to guide syntactic retrieval operations. However, this conclusion would be premature on the basis of these data alone. These studies largely investigated configurations where there was only one grammatically licit position that could serve as the target of the retrieval. For instance, when the verb initiates the retrieval for the clefted object filler in (1), *wh*-feature cues could unambiguously select the filler as the target of retrieval. For this reason, they provide no direct test of whether syntactic position information plays a role in helping the retrieval mechanism to distinguish between multiple, syntactically accessible targets in different syntactic domains.

A potential exception is Martin and McElree (2011), who investigated the processing of sluiced sentences as in (3):

- Distant VP: Michael drank coffee and typed something, but he didn't tell me **what**.
- Recent VP: Michael typed something and drank coffee, but he didn't tell me **what**.    (3)

Martin and McElree hypothesized that the processing of the sluiced *wh*-phrase *what* requires comprehenders to retrieve an antecedent VP from memory, which is then used to construct the elided clause (i.e., the IP) at the sluice site. Martin and McElree manipulated the distance between the antecedent VP (*typed something* in 3) and the sluiced *wh*-phrase. In addition, they attempted to manipulate the size of the antecedent search set by manipulating whether a competitor VP was present in a coordination structure (*drank coffee* in 3). Nonetheless, they observed that only accuracy, not processing speed, was negatively impacted by the presence of multiple VP antecedents. They additionally observed that the recency of the antecedent VP only impacted retrieval accuracy. They argued that this pattern of results provided a strong data point in favor of content-addressable retrieval operations over syntactically structured search operations. However, because the two candidate antecedents were coordinated, this study may not have effectively manipulated the syntactic locality of the antecedent VP. Both potential antecedents were in a structurally similar position in the preceding clause. For this reason, this study leaves unresolved the question of how structural locality impacts retrieval.

### The current study

In the present study we evaluate the Local Search hypothesis by investigating the processing of the Mandarin Chinese long-distance reflexive *ziji*. The anaphor *ziji* is an example of the cross-linguistically well-attested class of long-distance reflexives, reflexive pronouns that may be bound outside of their local clause. Thus, unlike the English reflexives *himself* and *herself, ziji* does not require that its antecedent be in the same clause, as seen in (4), where subscript indices are used to indicate possible coreference:

*Zhangsan*_j_ *shuo Lisi*_i_ *nongshang-le ziji*_i/j_
Zhangsan says Lisi harm-perf self
“Zhangsan says that Lisi harmed him/herself”    (4)

In (4), it can be seen that *ziji* may be bound either by the local subject *Lisi* or the matrix subject *Zhangsan*. Like many long-distance reflexives, *ziji* imposes a number of constraints on potential antecedents (Büring, 2005; Huang et al., 2009). There are significant syntactic constraints placed on antecedents: they must be subjects whose clausal projection dominates the clause that contains *ziji* (Huang and Liu, 2001). In addition to these syntactic constraints, there are a number of discourse-pragmatic constraints on the use of *ziji*. Antecedents must be animate and sentient, and must be prominent in the current discourse (Xue et al., 1994; Huang and Liu, 2001). In the absence of an appropriate antecedent in the immediate sentential context, *ziji* has been claimed to refer to the speaker, presumably as a reflex of the prominent discourse status that is automatically afforded to the speaker (Kuno, 1972; Huang and Liu, 2001). Though there are ongoing debates about the exact nature of *ziji*'s licensing conditions (Huang et al., 2006), it is uncontroversial that resolving the antecedent-anaphor dependency requires the comprehender to systematically exclude structurally unacceptable referents from consideration.

Although *ziji* can in principle take either local or long-distance antecedents, previous research suggests that there is a preference for local antecedents over more distant antecedents in online comprehension. For example, Li and Zhou (2010) provide ERP evidence that long-distance binding of *ziji* elicits a larger P300/600 response relative to local or ambiguous binding of *ziji*, suggesting greater processing difficulty associated with recovering long-distance interpretations. In addition, cross-modal priming studies have shown that probes associated with local antecedents are recognized more quickly than probes to long-distance antecedents upon encountering *ziji* (Gao et al., 2005; Liu, 2009). Chen et al. (2012) also present self-paced reading evidence that comprehenders read local *ziji*-antecedent dependencies more quickly than long-distance dependencies. These studies establish a preference for local binding over long-distance binding in comprehension, but without any direct time course evidence it is unclear whether this preference reflects a difference in retrieval speed or retrieval accuracy for local antecedents.

In this study we investigate the processing of local and long-distance interpretations of *ziji* as in (5). We take the embedded and matrix clauses in (4) to constitute distinct syntactic domains for the purposes of finding z*iji*'s antecedent.

- *Zhangsan*_i_ *shuo fengbao*_j_ *hai-le ziji*_i/j_
  Zhangsan says storm harm-perf *ziji*
  “Zhangsan said the storm harmed him.”
- *Xiaoshuo*_i_ *shuo Zhangsan*_j_ *hai-le ziji*_i/j_
  novel says Zhangsan harm-perf *ziji*
  “The novel said Zhangsan harmed himself.”    (5)

Because *ziji* requires an animate and sentient antecedent, only *Zhangsan* in (5a,b) is a grammatically licensed antecedent. Of critical interest is the long-distance configuration (5a), where the local subject is inanimate and thus semantically inappropriate as an antecedent for *ziji*. The critical empirical question in this comparison is whether comprehenders will show delayed access to the matrix antecedent in (5a). If the match on semantic cues outweighs the effect of dependency locality, and if it grants reliable direct access to the matrix antecedent, then the only difference between (5a) and (5b) should be the amount of time the antecedent has decayed in memory. Previous findings show that decay alone does not impact the speed of retrieval (see e.g., McElree et al., 2003). Thus, if semantic cue match outweighs the effect of locality in this configuration, we predict no difference in retrieval speeds between local and long-distance interpretations of *ziji*. Instead, we should see only a difference in processing accuracy between the two configurations in (5), such that local antecedents are more accurately retrieved.

The Local Search hypothesis, however, does predict a difference in retrieval speeds. If the parser initially uses cues that limit retrieval to the local clausal domain, then on a significant portion of trials the comprehender should misretrieve the local subject in (5a) despite its poor fit to *ziji'*s semantic cues. This would require the comprehender to engage costly reanalysis processes to recover the more distant antecedent, leading to slowed retrieval times in (5a). In Experiment 1, we used a variant of the SAT technique known as multiple response SAT (MR-SAT) to estimate the speed of processing *ziji* in these two configurations to determine whether local and long-distance *ziji* dependencies are associated with different retrieval speeds.

## Experiment 1

Experiment 1 employed the multiple-response speed-accuracy tradeoff procedure (MR-SAT; Wickelgren et al., 1980) to estimate the time course of retrieving an antecedent for *ziji* in sentences such as (5). MR-SAT is an attractive technique to use in studying language comprehension because it dissociates processing speed from processing accuracy (McElree, 2000; McElree et al., 2003; Foraker and McElree, 2007; Martin and McElree, 2008, 2009, 2011). In a MR-SAT paradigm, participants are required to make acceptability judgments at pre-specified response latencies. This provides a measure of how accuracy grows over time, and thus provides a direct measure of the time course of processing. In contrast, single RT paradigms are limited in how informative they are about time course of processing. Because participants can trade speed and accuracy in many standard judgment tasks (Wickelgren, 1977), merely estimating a point RT per condition (or a single RT/accuracy pair) can obscure differences between the probability of successfully completing a process and the speed with which that process reaches completion. In contrast, the full time course summarized in an SAT function allows the researcher to separately estimate the speed and the accuracy of memory retrieval. In the present case, we are concerned with the nature of any difficulty observed with non-local *ziji* interpretations as in (5a). Prior work suggests that retrieval difficulty associated with temporal decay or linear distance is associated with a decrease in retrieval accuracy, rather than retrieval speed (McElree, 2000; McElree et al., 2003; Foraker and McElree, 2007; Martin and McElree, 2008, 2009). Based on these results, we do not expect to observe differences in retrieval speed purely as a function of decay or recency.

### Method

#### Participants

Twenty college students from Beijing Normal University participated in the experiment. Data from 3 participants were excluded for reasons that are detailed below. The remaining 17 participants included 10 females, and had a mean age of 23.5 years. Each participant completed six 1-h experimental sessions spaced at least a day apart, in addition to a 1-h practice session for familiarization with the MR-SAT procedure. All participants were native Mandarin Chinese speakers and had normal or corrected-to-normal vision. Following an IRB-approved protocol, all participants gave informed consent and were paid 35 RMB per hour for their participation in the experiment.

#### Materials

Our critical experimental materials were Mandarin sentences that contained a main clause verb that selects for a sentential complement (e.g., “*biaoshi*,” say). The embedded complement clause was always transitive, and the embedded object was always the sentence-final word. Two features of the stimuli were manipulated orthogonally, in a crossed 3 × 3 experimental design. One was the position of a syntactically prominent animate subject; it was either the subject of the main clause (*long distance animate)*, the subject of the local (embedded) clause (*local animate*), or not present (*no antecedent*). In addition we manipulated the form of the embedded object NP, which was either the long-distance reflexive *ziji*, a contextually plausible definite NP, or a contextually implausible definite NP.

Four of the nine resulting conditions formed the critical experimental conditions (Table 1). Based on the position of the animate subject, *ziji* either took a long-distance antecedent (Long-distance animate*, ziji* condition) or a local antecedent (Local animate, *ziji* condition). In the control conditions *ziji* was replaced with a full NP that was a plausible object of the embedded verb (e.g., *the batsman*). The inclusion of these control conditions helps to ensure that any differences in processing speed or accuracy observed in the critical *ziji* conditions are specific to retrieval processes associated with *ziji*, rather than other properties of the sentence frame. In the critical experimental conditions, sentences were acceptable across all four conditions.

**Table 1:** **Summary of the critical conditions in Experiment 1**.

**Condition**	**Example**
Long-distance animate, *ziji*	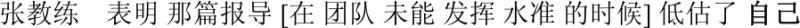 Coach Zhang say [that report (when team not perform well-time) underestimate **ziji**] “Coach Zhang says that that report underestimated ***self*** when the team was doing poorly.”
Local animate, *ziji*	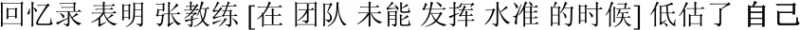 Auto-biography say [coach Zhang (when team not perform well-time) underestimate **ziji**] “The auto-biography says that coach Zhang underestimated ***self*** when the team was doing poorly.”
Long-distance animate, control	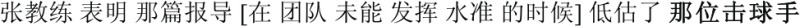 Coach Zhang say [that report (when team not perform well-time) underestimate **that** **batsman**] “Coach Zhang says that that report underestimated ***the batsman*** when the team was doing poorly.”
Local animate, control	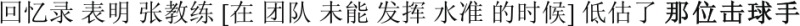 Auto-biography say [coach Zhang (when team not perform well-time) underestimate **that batsman**] “The auto-biography says that coach Zhang underestimated ***the batsman*** when the team was doing poorly.”

In the local *ziji* and the corresponding control conditions, the main clause subject NP was always an inanimate noun that described a form of written or spoken media (e.g., *book, documentary, memo*) to ensure compatibility with the meaning of the main clause verbs (e.g., *say*) while being an unacceptable antecedent of *ziji*. None of the inanimates used in any position could be construed metonymically; metonymic interpretations of inanimates (i.e., *the newspaper* being used to refer to the employees of the newspaper) may be used as antecedents for *ziji*. In order to ensure that participants do not have ceiling performance in our task (McElree, 2006), a temporal adverbial clause was interpolated between the embedded subject and the embedded verb. In all conditions, an animate NP was used as the subject of the temporal adverbial phrase. However, since it occupied a position that is not structurally higher than *ziji*, it is not a grammatical antecedent for *ziji*.

In addition to these critical four conditions, the implausible object conditions contained a contextually implausible embedded object (e.g., “The auto-biography says that the coach underestimated *the glasses* when the team was doing poorly.”) and the no animate conditions did not contain an animate NP in either the matrix or embedded subject position (e.g., “The auto-biography says that the report underestimated *ziji* when the team was doing poorly.”). These extra conditions provided unacceptable counterparts to the critical conditions, either because of a local implausibility, or because *ziji* did not have an antecedent available. There were two reasons for including these additional conditions, despite the fact that they were not part of the primary experimental manipulation. First, they provided unacceptable sentences that could be used in *d*-prime scaling. More importantly, the inclusion of the implausible object and no antecedent *ziji* conditions ensured that within the experiment neither the presence of *ziji* nor the acceptability of the sentence was predictable from the sentence context. Because it is typical for a subject in an SAT experiment to see all conditions of an experimental item, the inclusion of these additional conditions was critical to ensure that participants could not use familiarity with the sentence context to anticipate their response in advance of the sentence final word.

Forty sets of the 9 sentence types (5 acceptable and 4 unacceptable) were generated. The resulting 360 sentences were equally distributed in 6 presentation lists, one for each of the 6 sessions, to minimize the repetition of content material within a session. Thus, across the six sessions, each participant saw each experimental item in each of its 9 conditions. Crucially, no two instances of *ziji* sentences from the same item set were presented within the same session. Within a session, each participant viewed 206 sentences, of which 60 were drawn from the current study. Since only one third of target sentences contained *ziji*, the critical *ziji* conditions comprised around 10% of all sentences within and across sessions. The order of presentation within a session was randomized.

#### Procedure

Stimulus presentation, timing, and response collection were all carried out on a personal computer using the Linger software (Rohde, 2003). Each trial began with a 500 ms fixation cross presented in the center of the screen. Each word appeared in the center of the screen for 400 ms, followed by 200 ms of blank screen. All words were presented using simplified Chinese characters, and the last word of each sentence was marked with a period (°). At the onset of the final word, a series of 18 auditory response cues (50 ms, 1000 Hz tone) was initiated. The cues occurred every 350 ms, and the final word of the sentence remained on the screen. Participants were asked to decide for each sentence whether it was an acceptable, coherent sounding sentence or not (in Mandarin: *tōngshùn he héshì*). Participants were trained to initially respond by pressing both response keys simultaneously to indicate an undecided response, and to respond at every tone. They were then trained to switch their response to either the “accept” or “reject” key as soon as they could. Importantly, they were also trained to modify their responses if their assessment changed. During the 1-h practice session, participants were told that some of the sentences were complex, but nevertheless were meaningful sentences, and explicit feedback was given about acceptable and unacceptable sentences in the experiment. Each participant performed six 1-h experimental sessions, and in each they saw one of the lists of materials. The order of lists was randomized across participants.

#### Data analysis

To derive the full time-course information, *d*′ scores were calculated by comparing an acceptable and an unacceptable condition at each of the response tones. The resultant series of *d*′-values at each time point *t* was fit using a shifted exponential function:

(6) $$\begin{array}{l} d^{\prime }=\lambda \left(1-e^{-\beta (t-\delta )}\right),t>\delta ,\\ d^{\prime }=0\;,otherwise \end{array}$$

The SAT function in Equation (6) describes the growth of accuracy over time using three parameters: *asymptote* (λ), *rate* (β), and *intercept* (δ). By regressing the non-linear SAT function against the time course data collected in the experiment, we may make inferences about the effect of experimental manipulations on each of the parameters. The initial period of chance performance is described by the intercept parameter (δ), which indicates the point at which the SAT function departs from chance performance (0 in *d*′ units). The next portion of the function is characterized by a period of increasing accuracy; the rate of growth in this portion of the SAT function is described by the rate parameter (β). The last portion of the function reflects terminal accuracy in the behavioral judgment, and it is reflected in the asymptote parameter of the SAT function (λ). The intercept and the rate together index the speed of the process, while the asymptote indexes the terminal accuracy of the process. The processing speed may also be evaluated by considering a composite measure known as the *speed* of the SAT function (β^−1^ + δ). By parameterizing the SAT function in this way, we can separately estimate the speed of processing (as reflected in the intercept, rate, or speed measures) and the accuracy of processing (as reflected in the asymptote). Differences in the intercept or rate parameters indicate a difference in processing speed between two conditions; differences in the asymptote parameter indicate a difference in processing accuracy.

*d*′ is the standard measure of discrimination (assuming equal-variance Gaussian distributions): *d*′ = Φ (hits) − Φ (false alarms) (Macmillan and Creelman, 2004; Φ represents the inverse of the cumulative distribution function of the standard normal). However, in the models reported below, we only report a *pseudo d*′ measure that does not correct for the false alarm rate [*d*′ = Φ(hits)]. We adopted this analysis because the somewhat high acceptability of the no antecedent condition (see below) made it inappropriate for the construction of a discriminative *d*′ measure. For reference, a pseudo *d*′ score of 2.5 represents perfect performance in our experiment, and a pseudo *d*′ score of 0.84 represents a hit rate of 0.80.

It is important to note that our pseudo *d*′ measure does not correct for any response bias that participants may have. In this respect, our analysis differs significantly from the approach adopted in previous SAT work, which has generally used *d*′ as the dependent measure to ensure that any time course differences are not simply due to differences in response bias across conditions. However, we note that our critical conditions constitute a 2 × 2 crossed factorial design (presence of *ziji* by position of animate antecedent). This design allows us to account for any response bias introduced by two major features of our stimuli: the configuration of the sentence prior to the critical final word, and the presence of *ziji* in final position. If response bias varies as a function of the sentence context, then this bias should be shared by both *ziji* and control conditions. Likewise, if there is response bias associated with a sentence final *ziji*, as opposed to a sentence final lexical NP, then this bias should be shared by both *ziji* conditions. Thus, any interactions of *ziji* and position of the animate subject in our design cannot be the result of response bias introduced by either of these two configurations.

Data analysis proceeded in two steps: a model selection analysis and a parameter estimation analysis (Liu and Smith, 2009). In the model selection analysis, the best fit SAT model was determined using the adjusted *R*^2^-statistic (in Equation 7) using a hierarchical model-testing scheme over the averaged data, an approach pursued in prior work on SAT in sentence comprehension (McElree, 2000; McElree et al., 2003; Foraker and McElree, 2007; Martin and McElree, 2008, 2009). However, we note that for multiple-response SAT, determining the number of independent data points *n* is not a trivial problem, because of the lack of independence between responses on any trial. Because of the uncertainty concerning the number of truly independent data points that underlie any one MR-SAT function, it is difficult to straightforwardly apply model fitting metrics such as adjusted *R*^2^, the AIC, and the BIC. In the parameter estimation analyses, only fully saturated models that allow all parameters to vary by condition are considered, and any differences between the critical conditions on the parameters of interest are assessed using familiar hypothesis testing measures over individual parameter estimates. This analysis follows the recommendations of Lorch and Myers (1990) for dealing with regression analyses in the context of a repeated measures experiment. In order to obtain parameter estimates, we used the R statistical computing environment to fit non-linear regressions of the SAT function Equation (6) against the pseudo *d*′ score (see McElree and Griffith, 1998; McElree, 2000; McElree et al., 2003; Martin and McElree, 2008, 2009, 2011). We used the nls() function with an adaptive non-linear least squares algorithm (Dennis et al., 1981) to determine the least squares fit of the SAT function to the data.

(7) $$R^{2}=1-\frac{\sum_{i\;=\;1}^{n}{\left(d_{i}-{\hat{d}}_{i}\right)}^{2}/(n-k)}{\sum_{\;i\;=\;1}^{n}{\left(d_{i}-\bar{d}\right)}^{2}/(n-k)}$$

Prior to modeling the *d*′ scores, analysis was performed on empirical pseudo *d*′ measures by participants. This was obtained by taking the average rate of acceptance over the last four response points in each condition to determine the empirical hit rate, and calculating *d*′ as described above. Hit rates that reflected perfect performance were smoothed by subtracting 0.0125 from the hit rate [1/(2N) smoothing (Macmillan and Creelman, 2004)].

Where appropriate, behavioral measures and parameter estimates from the SAT function in Equation (6) were further analyzed by entering them into a 2 × 2 repeated-measures ANOVA crossing dependency type (*ziji* vs. *control*) and the position of the animate argument (*long-distance* vs. *local*).

Of the twenty participants run, data from two participants were excluded due to unreliable dynamics estimates. The empirical *d*′ scores from these participants appeared better fit by a sigmoidal rather than exponential function, leading to unrealistically large and unreliable differences in the critical conditions in the crucial intercept and rate parameters when fit with the SAT function in Equation (6). The data from one further participant were rejected due to lower than 60% correct responses on both critical *ziji* conditions. These participants' empirical *d*′ were not included in any analyses below.

### Results

#### Accuracy and empirical d′ analysis

For the four critical experimental conditions, acceptance rates were high. Average acceptance was 87% for long-distance *ziji* conditions and 83% for local *ziji* conditions. The rates of acceptance for the long distance and local control conditions were 91 and 88%, respectively.

In contrast, the average acceptance was 47% percent for no antecedent *ziji* conditions, and the unacceptable control conditions each had an average acceptance rate of 2%. In addition, the rate of acceptance of the no antecedent acceptable control condition was 92%.

Table 2 presents the mean empirical pseudo *d*′ for *ziji* and control conditions. The data were analyzed using a 2 × 2 repeated-measures ANOVA with dependency type and animate position as within-participant factors. This analysis revealed a marginal main effect of position of animate argument [*F*_(1, 16)_ = 3.6, *p* < 0.1], as well as a marginal effect of dependency type [*F*_(1, 16)_ = 4.3, *p* < 0.1]. The interaction of animate position and dependency type was not significant (*F* < 0.1). However, planned comparisons between the long-distance and local conditions within *ziji* and control conditions did not reveal any reliable effects [*ziji*: *t*_(16)_ = 1.02, *p* = 0.32; control: *t*_(16)_ = 1.6, *p* = 0.12].

**Table 2:** **Mean empirical pseudo *d*′-values, obtained by averaging accuracies over final four response latencies**.

	***ziji***	**Control**
Long-distance animate	1.26 (±0.12)	1.53 (±0.12)
local animate	1.11 (±0.13)	1.33 (±0.13)

*By-participant standard error in parentheses*.

#### Time course analysis

Competitive fits of the shifted exponential function in Equation (6) were conducted to assess differences in asymptotic accuracy, rate, and intercept across conditions for each participant. Model fits were conducted separately for control and *ziji* conditions. Because the empirical *d*′ analysis revealed only marginal differences between conditions in accuracy, it is not clear whether competitive model fits are justified in allowing the asymptote parameter to vary freely between conditions. In light of this, we fitted two SAT functions to each data set: one model whose asymptote parameter was fixed to the empirical pseudo *d*′ obtained by averaging over the final four response latencies, and one where the asymptote parameter was allowed to vary. We report results from the free parameter models, but we note that fitting the models with fixed asymptotes did not yield qualitatively different results.

**Figure 1:**
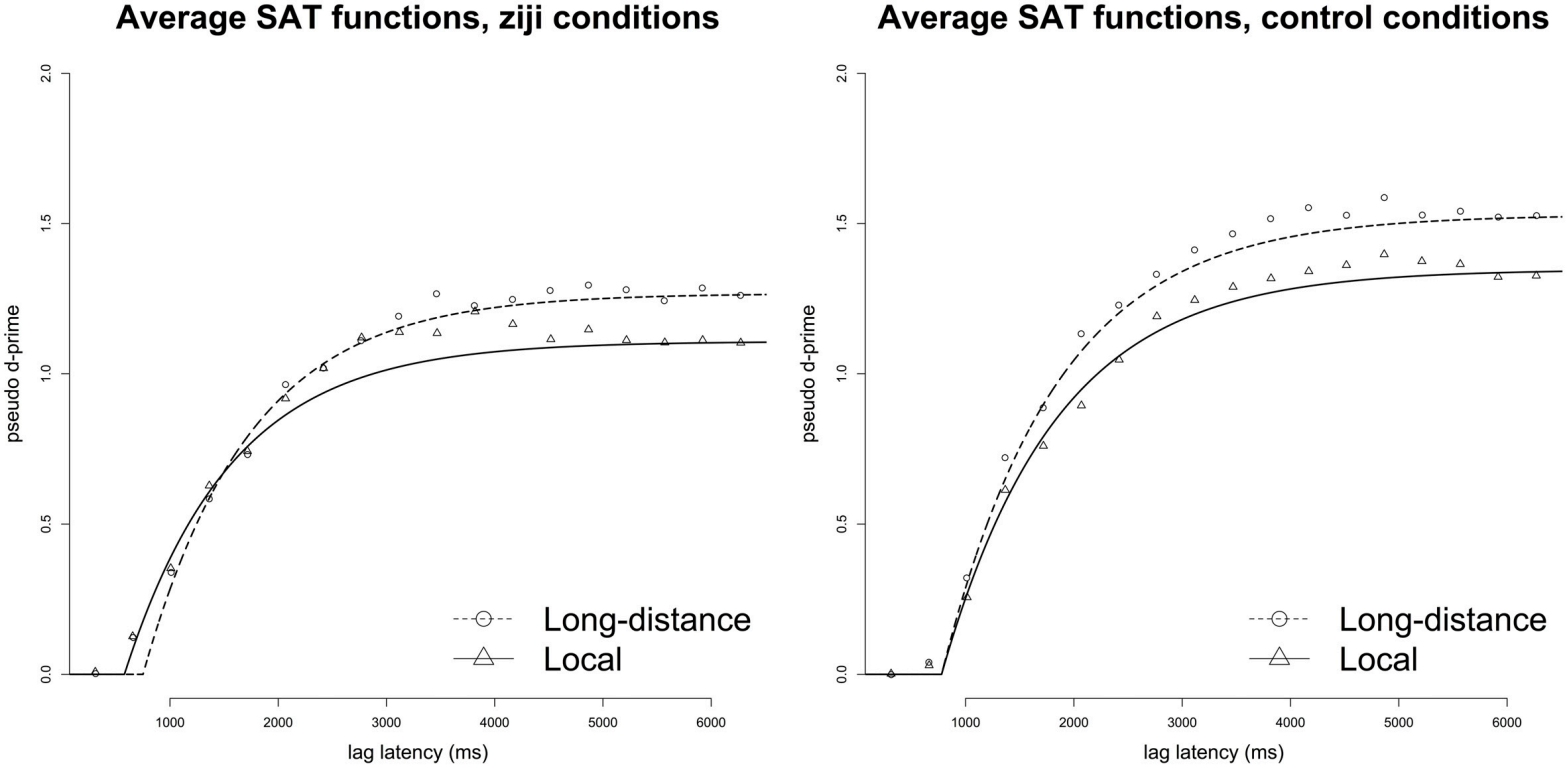
**Time course data (points) and best-fit SAT functions (lines) to average pseudo *d*′ scores in Experiment 1, for *ziji* and control conditions**.

Model-fitting analyses pitted nested models against each other on adjusted *R*^2^ Equation (7), following McElree et al. (2003) and Liu and Smith (2009). Fits to the across-participants average for the critical *ziji* conditions revealed a small advantage for models that allocated separate intercept parameters (δ) for local and long-distance conditions (2λ-1β-2δ, *R*^2^:0.986) and models that models that posit separate rate (β) parameters (2λ-2β-1δ, *R*^2^:0.985) over models that posited shared rate and intercept parameters for the two conditions (2λ-1β-1δ, *R*^2^:0.982). This difference reflected a small rate advantage for local *ziji* condition over long-distance *ziji* condition (LD β: 0.96 s^−1^, Local β: 1.26 s^−1^; LD δ: 0.75 s, Local δ: 0.58 s). These models were in turn a better fit to the data than any model that contained only a single asymptote for both conditions (max *R*^2^ = 0.974). Control conditions showed no improvement in fit for additional rate or intercept parameters (2λ-1β-1δ, *R*^2^:0.996; 2λ-2β-1δ, *R*^2^:0.996; 2λ-1β-2δ, *R*^2^:0.996). The average data for *ziji* and control conditions, along with best-fit models on the adjusted *R*^2^ metric, are presented in Figure 1.

Of critical interest is whether the fits to the average data reflect a reliable trend across individuals. It is possible that the SAT function reflected in the average is not in fact representative of a pattern observed in any individual subject (Liu and Smith, 2009), and in the present case, there was a very small difference between models with different dynamics parameters and those without. In order to assess the reliability of parameter estimates across participants, each individual's *d*′ data were fit with the SAT function separately for each of the four critical conditions. As before, fits were conducted both with fixed and free asymptote parameters, and these two types of models did not yield qualitatively different results. Thus, we report only the results from models with free asymptote parameters. Fits to individual participants revealed differences in whether dynamics differences between the critical conditions were reflected in the SAT function's rate, its intercept, or both. Because of these differences, here we additionally present and analyze the speed measure (β^−1^ + δ). This composite measure allows us to quantify processing speed in a uniform way across individuals in the face of this variation. The results of this analysis for the critical *ziji* conditions are presented in Table 3.

**Table 3:** **By-subject and average parameter estimates for critical ziji comparisons, along with average parameter estimates for control comparisons**.

	**LD *R*^2^**	**Loc *R*^2^**	**Asymptote (λ, *d*′)**		**Rate (β, *s*^−1^)**		**Intercept (δ, *s*)**		**Speed (β^−1^ + δ, *s*)**	
			**LD**	**Local**	**LD**	**Local**	**LD**	**Local**	**LD**	**Local**
Average	0.99	0.98	1.30	1.15	0.95	1.27	0.72	0.74	1.76	1.53
S1	0.98	0.96	0.95	0.72	3.51	15.00	0.29	0.30	0.58	0.37
S2	0.75	0.94	1.66	1.55	2.81	3.90	0.93	0.89	1.29	1.15
S3	0.97	0.98	2.00	0.95	0.72	1.38	0.88	0.96	2.27	1.69
S4	0.99	0.86	0.95	1.75	3.87	1.95	0.65	0.68	0.90	1.20
S5	1.00	0.98	0.68	0.30	0.86	15.00	0.28	0.54	1.44	0.61
S6	0.99	0.98	0.93	0.59	1.36	3.57	1.08	1.25	1.81	1.53
S7	0.95	0.96	1.89	2.21	2.08	1.77	0.57	0.58	1.05	1.15
S8	1.00	0.93	1.05	2.26	3.81	0.97	1.58	1.26	1.84	2.29
S9	0.91	0.96	1.76	0.44	1.09	4.65	1.66	1.92	2.58	2.14
S10	0.97	0.97	0.57	0.99	1.67	3.27	1.18	1.44	1.78	1.75
S11	0.98	0.99	1.65	0.97	0.60	1.49	0.15	0.28	1.81	0.95
S12	0.94	0.87	2.10	1.64	0.89	1.84	1.65	1.64	2.77	2.18
S13	1.00	0.99	0.40	0.76	1.26	1.33	1.73	0.99	2.52	1.74
S14	0.99	0.99	0.77	0.48	0.87	1.60	0.82	0.26	1.96	0.88
S15	0.98	0.92	1.38	1.71	2.14	1.23	1.00	1.19	1.47	2.00
S16	0.99	0.99	1.37	0.62	1.06	1.14	0.77	0.76	1.71	1.64
S17	0.98	0.99	2.03	1.67	1.01	3.32	0.60	0.81	1.59	1.11
Control	0.99	0.99	1.57	1.39	0.97	0.91	0.77	0.79	1.80	1.89

The individual parameter estimates were entered into a 2 × 2 repeated-measures ANOVA with dependency type and animate position as within-participant factors. ANOVAs revealed an interaction of dependency type and animate position both for rate parameters β [*F*_(1, 16)_ = 4.8, *p* < 0.05] and for the composite speed measure β^−1^ + δ [*F*_(1, 16)_ = 8.2, *p* < 0.05]. In addition, there was a main effect of dependency type on speed measures [*F*_(1, 16)_ = 6.1, *p* < 0.05] and on rate parameters [*F*_(1, 16)_ = 3.6, *p* < 0.1], reflecting faster processing of *ziji* conditions. Additionally, a significant effect of dependency type was observed for the asymptote parameter λ [*F*_(1, 16)_ = 4.9, *p* < 0.05]. There were no significant effects on the intercept parameter δ for either fixed or free asymptote models.

Planned pairwise comparisons revealed that these interactions were driven by differences in the *ziji* conditions in the speed measure β^−1^ + δ [*t*_(16)_ = −2.6, *p* < 0.05]. This analysis revealed only marginal effects on the rate parameter β [*t*_(16)_ = 1.9, *p* < 0.1]. There were no effects of antecedent position in the control conditions for either speed [*t*_(16)_ = 1.6, *p* = 0.12] or rate [*t*_(16)_ = −0.1, *p* = 0.89]. On average, the speed measure for local *ziji* condition was 294ms faster (95% CI: 52–538 ms) than long-distance *ziji* condition.

### Discussion

Both individual and average data suggest a time-course advantage for local *ziji* conditions compared to LD *ziji* conditions. In competitive model fits to the average time course data, there was a slight advantage for models that allocated different rate parameters to long-distance and local *ziji* conditions; no such difference was observed for control comparisons. An analysis of the parameter estimates for individual participants showed that for the critical *ziji* comparison, the local condition was processed significantly faster than the long-distance condition, as reflected in the rate parameter (β) and the composite speed measure (β^−1^ + δ). No difference was observed in control conditions. In ANOVA analyses of empirical *d*′ scores, there was no significant difference for either *ziji* or control comparisons in asymptotic accuracy.

#### Follow-up experiment

One unexpected finding in Experiment 1 was the high acceptance rate of the no antecedent *ziji* condition, which participants accepted on 47% of trials. It has been claimed that in the absence of an overt, syntactically prominent antecedent, *ziji* can refer to the speaker (Huang and Liu, 2001). However, post-experiment debriefing suggested that some speakers also interpreted *ziji* as coreferential with an implicit author of the inanimate main clause subjects such as *book* or *speech*. It is also possible that the subject contained in the temporal adjunct in our experimental sentences contributed to retrieval interference, and was misinterpreted as an antecedent for *ziji* on some trials. We conducted a follow-up experiment to determine the interpretations that comprehenders might assign to each of our three conditions.

The follow-up experiment used the three *ziji* conditions from Experiment 1. Twenty-four of the original 40 item sets were selected at random, and were distributed in a Latin Square fashion into three experimental lists. Each list was presented as a short questionnaire on the online experimental platform IbexFarm (Drummond, 2011). Each sentence was presented on the screen, and participants were instructed to choose their preferred interpretation of *ziji*'s antecedent from five options: the *main clause* subject, the *local* subject, the *interfering* subject contained inside the temporal adjunct, the *speaker* of the sentence, or *none*. When the main clause subject was inanimate (e.g., *book*), the main clause subject response option referred to the implicit author (e.g., *the book's author*).

Seventeen native Mandarin speakers were recruited via the Internet. The results are presented in Table 4. In order to test for differences across conditions in the proportion of responses, each response category was converted into a binary variable that was 1 if a response was in the category, and 0 otherwise. Each response category was analyzed using logistic mixed effects models with crossed random intercepts for subjects and items and random slopes of condition for subjects. Two Helmert contrasts were employed for the condition factor: a *locality* contrast that compared local and LD conditions, and an *antecedent* contrast that contrasted the no antecedent condition with the average of the LD and local conditions.

**Table 4:** **Average proportion of interpretations reported on critical ziji comparisons in the follow-up experiment**.

	**Long-distance**	**Local**	**Interfering**	**Speaker**	**None**
LD antecedent	0.87	0.06	0.03	0.00	0.04
Local antecedent	0.03	0.91	0.03	0.00	0.03
No antecedent	0.35	0.12	0.31	0.04	0.18

This analysis revealed that the *no antecedent* condition had significantly more *none* responses (β = 0.64, Wald's *z* = 3.4, *p* < 0.05) and *interferer* responses (β = 1.53, Wald's *z* = 6.1, *p* < 0.05) than the other two conditions. The LD condition had significantly more matrix subject responses than the local conditions (β = −3.5, Wald's *z* = −8.0, *p* < 0.05), and the local condition had significantly more embedded subject responses than the LD condition (β = 3.4, Wald's *z* = 8.8, *p* < 0.05). In addition, there was a significant effect of *antecedent* on embedded subject responses (β = −0.9, Wald's *z* = −5.2, *p* < 0.05), reflecting the low proportion of local subject responses in the no antecedent condition. No other effects were significant.

The follow-up experiment confirms that participants overwhelmingly select a structurally prominent, animate antecedent for *ziji* when there is one available. This replicates the judgments reported in the literature on Mandarin long-distance reflexives. Additionally, the results show that in the absence of a semantically appropriate and syntactically accessible antecedent, comprehenders nonetheless ultimately prefer a sentence-internal antecedent: the interfering subject is selected on 31% of trials, and on 35% of trials participants coerce an animate antecedent from the matrix subject.

### Discussion

The results of the follow-up experiment confirm that comprehenders prefer to select syntactically prominent, animate antecedents for *ziji* in our materials. The results of Experiment 1 show that comprehenders are measurably slower to access long-distance antecedents for *ziji* than local antecedents. The fact that dependency distance impacted retrieval speed in our SAT experiment contrasts with previous SAT findings. Previous work on SAT in language comprehension suggests that distance does not affect the dynamics parameters in the SAT function (McElree, 2000; McElree et al., 2003; Foraker and McElree, 2007; Martin and McElree, 2008, 2009). This makes it unlikely that the faster access times we observe to local antecedents reflect a simple effect of temporal distance or recency.

Another way that long-distance and local antecedent configurations differ is in the type of interference contributed by the semantically inappropriate antecedent. In long-distance conditions, the semantically inappropriate antecedent intervenes between the target antecedent and the anaphor, and so generates retroactive interference (RI) in the process of retrieving the target antecedent. Conversely, in local antecedent configurations, the long-distance antecedent precedes the target, and so generates proactive interference (PI) that may disrupt the anaphor's retrieval of its antecedent. The difference in the type of interference created by the semantically inappropriate antecedent may be critical: Öztekin and McElree (2007) observed that in recognition memory tasks, the presence of PI has an effect on retrieval dynamics, leading to slower retrieval times. However, recent SAT work has directly investigated the effects of PI and RI on retrieval processes in language comprehension (Van Dyke and McElree, 2011). Van Dyke and McElree (2011) suggest that RI contributes more difficulty in dependency completion in sentence comprehension than does PI, but crucially, they show that the type of interference (PI/RI) does not impact retrieval speeds in multiple-response SAT. Instead, they observe only that RI configurations lower asymptotic accuracy relative to PI configurations. In light of these results, it appears unlikely that the speed differences that we observed were due to the type of interference generated by the inappropriate antecedent.

## A model of the local search hypothesis

We have suggested that neither recency alone nor the type of interference (RI/PI) was the source of the observed differences in retrieval times in Experiment 1. Instead, we argue that these results suggest that comprehenders consider or misretrieve the local subject position when the target antecedent is syntactically distant, which then leads to slowed retrieval of long-distance antecedents. We propose that this arises because locality outweighs semantic cues when retrieving an antecedent for *ziji*. There are two potential explanations of this locality effect in our data. According to the Local Search hypothesis, this effect reflects the use of cues that restrict retrieval operations to a local syntactic domain. However, it is possible that this locality effect reflects more misretrievals of a semantically inappropriate local subject simply because it has a relatively high resting activation. There are two reasons to suspect that the local subject might have higher resting activation prior to reaching the anaphor *ziji*. First, it is more recent, and so will have undergone less temporal decay. Second, the embedded subject forms a dependency with the verb that precedes *ziji*. The process of retrieving the subject to form this dependency may boost the embedded subject's resting activation prior to encountering the anaphor.

To distinguish between a Local Search account and an account that attributes the slowed processing of LD *ziji* to the heightened activation of the local subject, we formalize the predictions of both accounts with a simple quantitative model of the antecedent retrieval process for *ziji*. Our model incorporates the declarative memory component of the ACT-R framework (Anderson and Lebiere, 1998), which implements a direct access cue-based retrieval process that is subject to temporal decay and retrieval interference. An attractive feature of this model is that it has been used in a number of successful models of cue-based parsers (Lewis and Vasishth, 2005; Lewis et al., 2006; Vasishth et al., 2008). Our goal in modeling the antecedent retrieval process using ACT-R is to estimate the effect of a local search strategy on SAT retrieval dynamics above and beyond the effects of interference and decay.

We define our retrieval models in terms of the set of cues (the retrieval probe) used to retrieve an antecedent from memory. The *unrestricted* retrieval model limits the probe to item information only. In our implementation, this includes category identity (*NP*), a case feature (+*Nominative*), which serves to identify subjects, and an animacy feature (+*Animate*). The latter two cues implement the syntactic and semantic constraints on *ziji*'s antecedent. The *local search* retrieval model includes these features plus a feature (+*Local*) that distinguishes the local clause from other clauses. In terms of our stimuli, this feature is used to distinguish the embedded clause from both the matrix clause and the adjunct clause. This feature implements the core claim of the Local Search hypothesis: that the parser uses positional information to restrict search to the local syntactic domain at retrieval, creating a retrieval process that explicitly prioritizes retrieval within a local syntactic domain (here taken to be the local clause).

Our model assumes that the process of finding *ziji'*s antecedent involves a series of serially executed, cue-based retrievals from a content-addressable memory store (consistent with the processing assumptions of Lewis and Vasishth, 2005). Once an item is retrieved, it is evaluated as the antecedent of *ziji*. If the retrieved item is rejected as an antecedent for *ziji*, then the processor samples another potential antecedent from the linguistic context, without replacement. We assume that the processor samples antecedents in this way until an appropriate antecedent is found. Under this model, cue match, temporal decay, and interference all influence the average number of sampling operations that are required to recover the correct antecedent for *ziji*. The more sampling operations are executed during the retrieval of an antecedent, the slower the speed of the SAT function that tracks this process.

To fit this model to the empirical data, we first determine the probability of successfully retrieving the target antecedent on each successive sampling operation. We determine these probabilities by simulation, using the equations that define declarative memory in ACT-R. The ACT-R component of the simulations reported below was developed by Badecker and Lewis (2007) using the R programming language (R Core Team, 2013). Under this model, the parser retrieves the item in memory with the highest activation value, where activation is a function of the match to retrieval cues and the resting activation of all items in memory. Formally, the activation of a memory item *i* (*A_i_*) is the sum of its resting activation *B*_i_, the match between the item and each of the *J* retrieval cues in the probe (*S_j_*), and random noise (ϵ):

(8) $$A_{i}=B_{i}+\sum_{j}W_{j}S_{ji}+\epsilon$$

The weight associated with each retrieval cue *W_j_* is the total amount of goal activation available *G* divided by the number of retrieval cues. The resting activation of item *i* is a function of temporal decay (controlled by the decay parameter *d*) over all *M* intervals *t_m_* since the item was last retrieved or created:

(9) $$B_{i}=ln\left[\sum_{m}t_{m}^{-d}\right]$$

The match of an item *i* to the retrieval probe is the sum of a weighted associative boost for each cue *S_j_* in the retrieval probe that matches the features of item *i*. The weight of a feature *W_j_* is assumed to be equal across all cues in the probe. The associative boost that a given cue adds to an item it matches is reduced by the *fan* of that cue, or the number of items in memory that match that cue:

(10) $$S_{ji}=S-\ln(fan_{j})$$

Lastly, a small amount of stochastic noise is added to every item's activation level. On any given trial a noise value is drawn from a logistic distribution with a mean of zero and a variance that is controlled by a noise parameter *s*.

(11) $$\epsilon \sim logistic(0,\sigma^{2})$$

(12) $$\sigma^{2}=\frac{\pi^{2}}{3}s^{2}$$

For all predictions reported below, we simulated the model's predictions on a range of parameter settings, and report the mean predicted values across all parameter settings (following the approach in Dillon et al., 2013). Our choices of possible parameter settings were based on the settings reported in Lewis and Vasishth (2005)^1^. One exception was the scaling parameter *F*, which was set to yield a mean retrieval time of 90 ms. This was chosen because it provided a close fit to the estimated retrieval time of 85 ms in the SAT paradigm found by McElree et al. (2003). The times between the creation of antecedent representations and the retrieval associated with *ziji* were calculated directly from the experimental presentation parameters. In addition, an intermediate retrieval of the local subject at the embedded verb was simulated, which provided a boost in the embedded subject's resting activation prior to the point when *ziji* was encountered.

For both retrieval models, the probability of retrieving the target and each of the distractor NPs under these conditions was estimated using Monte Carlo simulation and averaging across all parameter settings considered. From this distribution we simulated the average number of sampling operations necessary to recover the target antecedent for both retrieval models. The resulting distributions are presented in Table 5. Under local search models, the local target is reliably retrieved after only one sampling operation on 56% of trials, whereas the modal number of sampling operations required to access the long-distance antecedent under the search model is 3 (occurring on 55% of trials). In the unrestricted search models, there is a lower probability of success with a single retrieval: local antecedents are retrieved on the first trial in 35% of trials for unrestricted models, and long distance antecedents are retrieved on 27% of trials. The lower probability of success for unrestricted models reflects the additional interference from the syntactically illicit distractor NP that occurs without positional cues to retrieval. On unrestricted models the number of sampling operations necessary to recover the target antecedent does not appear to differ substantially for local and long-distance antecedents. This pattern suggests that the increased resting activation of the local subject does not by itself lead to a substantially increased rate of retrieval errors during the retrieval of a long-distance antecedent. Instead, the search model results suggest that a semantically inappropriate local subject is most likely to be misretrieved when the search probe contains positional cues that select the local subject.

**Table 5:** **Probability distribution over the average number of sampling operations necessary to recover ziji's antecedent for the critical experimental conditions, for each of the candidate retrieval models**.

***P* (number of samples = *X*)**	**1**	**2**	**3**
LD antecedent, unrestricted	0.270	0.385	0.345
Local antecedent, unrestricted	0.353	0.437	0.210
LD antecedent, local search	0.190	0.258	0.552
Local antecedent, local search	0.560	0.351	0.089

Next, we calculated the distribution of finishing times for the search process under the serial sampling model we have proposed. We simulated the distribution of finishing times for a retrieval process with *n* sampling iterations by simulating the sum of *n* retrievals from the ACT-R model given above. For retrievals beyond the first, an additional 50 ms was added, reflecting the additional processing necessary to evaluate the retrieved item^2^. In ACT-R, the retrieval latency *T*_i_ is a function of activation and a scaling parameter *F* (see Footnote 1):

(13) $$T_{i}=Fe^{-Ai}$$

Inspection of the resulting finishing time distributions showed that they were well-fit by gamma distributions. Therefore, we modeled the overall predicted finishing time distribution for a given retrieval model as a mixture of gamma distributions, with each component reflecting the distribution of finishing times for a process with *n* sampling iterations. The mixing probabilities on each component were provided by the distribution in Table 5. With this mixture distribution, we could then follow the modeling approach advanced by McElree (1993). To do this, we used the resulting mixture to model the probability that the retrieval process will have completed by any time *t* as the cumulative distribution of this mixture, offset by a constant base encoding time δ (McElree, 1993):

(14) $$\begin{array}{l} P\left(T\leq t\right)=\frac{\beta^{\alpha }}{\left(\alpha -1\right)}\int_{0}^{t-\delta }e^{-\beta t^{\prime }}{t^{\prime }}^{\alpha -1}dt^{\prime }\\ \;t>\delta ,\text{ else }0. \end{array}$$

This cumulative distribution was then used to estimate the probability of responding with a hit at each time point *t*. This was calculated following the method described in McElree (1993). In particular, we assumed that all unfinished processes at time *t* contributed a hit 50% of the time, reflecting a guess on the part of the participant. We additionally assumed that on 5% of trials the target antecedent was rejected, leading to a miss response. The predicted proportion of hits at each time point was then transformed using the inverse cumulative normal distribution. Finally, the SAT function was fit to the predicted curves for each retrieval model and parameter setting, and the speed measure β^−1^ + δ was estimated for each predicted curve. We define the *locality advantage* as the predicted speed to access a long-distance antecedent minus the predicted speed to access a local antecedent, given a set of model parameters and a retrieval probe. The predicted locality advantages were calculated for both retrieval models, under all parameter settings. The predicted locality advantages were then compared to the empirical locality advantage in speed observed in Experiment 1.

**Figure 2:**
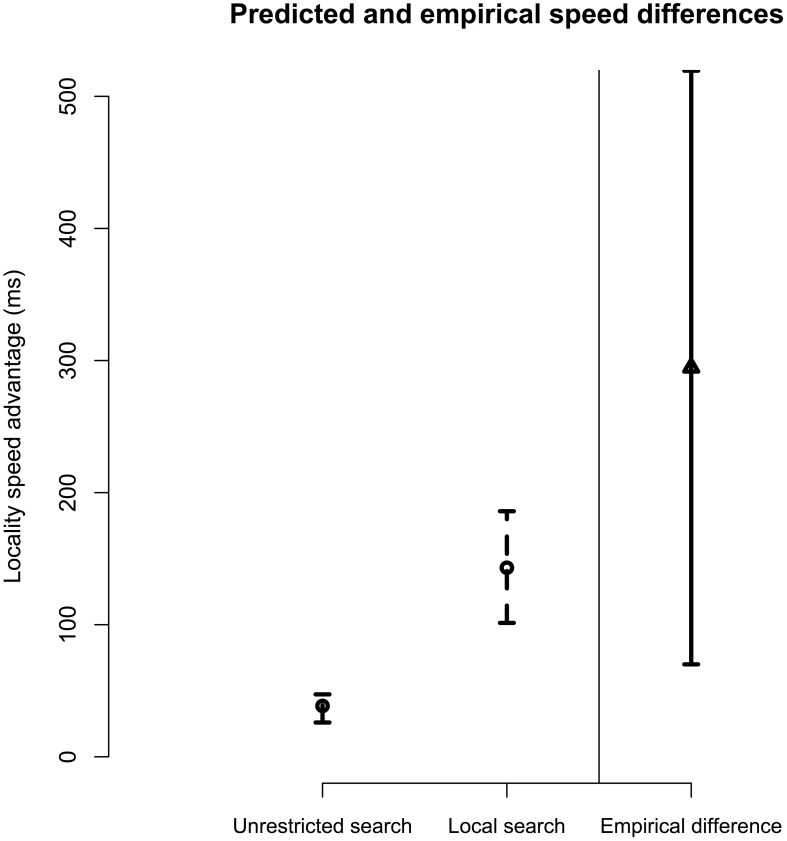
**Comparison of empirical speed advantage for local *ziji* antecedents and search model's predictions, given both unrestricted and local search retrieval probes**. For model predictions, error bars represent the 95% central range of predicted locality advantage over parameter settings. For empirical data, error bars represent 95% confidence interval of true speed advantage for local antecedents by participants.

Figure 2 provides a comparison of the empirical locality advantage with the predicted locality advantages for unrestricted and local search models. It can be seen that the local search model provides a good fit to the SAT data. On average, local search models predicted a locality advantage of 143 ms, approximately half of the observed empirical estimate of 294 ms from Experiment 1. However, the unrestricted search model predicts a much smaller speed advantage for local antecedents (39 ms). We tested the fit of each candidate retrieval model to the data by comparing the distribution of predicted locality advantage effects to the distribution of the mean locality effect estimated in Experiment 1. From these distributions, we calculated Bayes factors using the model comparison approach advocated by Gallistel (2009). This comparison gives 5:1 odds in favor of the local search model over the unrestricted model, providing “substantial” evidence in favor of the Local Search model (Jeffreys, 1961).

The modeling results suggest that the local search model provides a better explanation of our experimental data than does an account in which the locality advantage is simply attributed to the heightened activation of the local subject. We note that the model does confirm that an unrestricted model of antecedent retrieval does predict a small speed difference in the SAT function, due to the interaction of temporal decay, RI, and reactivation of the local subject prior to the anaphor. However, given the modeling assumptions here, these factors alone were not sufficient to allow the model to capture the findings of Experiment 1. By providing evidence against these plausible alternative explanations for the results of Experiment 1, the findings from the computational simulations lend additional support to the Local Search hypothesis.

## Discussion

### Summary of results

The current study presented time-course data from the MR-SAT paradigm on the processing of the Mandarin Chinese long-distance reflexive *ziji*. Non-linear regressions using the SAT function revealed that the parameters that describe the speed of processing (specifically, rate and speed) were significantly faster for sentences containing a local antecedent for *ziji* than for sentences with a long-distance antecedent for *ziji*. Control conditions without any anaphoric dependency showed no difference in speed or rate parameters. We observed only marginal differences in accuracy. Sentences with long-distance animate subjects were accepted at slightly higher rates for *ziji* and control conditions alike, and control conditions were accepted at a higher rate than *ziji* conditions. A follow-up experiment evaluated the interpretations that comprehenders assigned to *ziji* to a subset of our experimental materials. These results confirmed that participants overwhelmingly interpreted a local animate subject as the preferred antecedent for *ziji* when it was present, and likewise when there was a long-distance animate subject present. However, when there was no syntactically licit animate subject in the sentence, participants either rejected *ziji* as antecedentless, interpreted *ziji* as coreferent with an implicit possessor argument in the highest subject position, or interpreted it as coreferent with an animate subject embedded inside a temporal adjunct clause.

To aid in interpreting these data, we fit the predictions of two retrieval models to the SAT data. The Local Search model implemented a retrieval process that used positional syntactic cues to restrict retrieval to the local clause. The Unrestricted model used only item information to access potential antecedents. We showed that the Local Search model provided a closer fit to the empirical data than the Unrestricted model, using plausible parameter estimates.

### Locality in retrieval

The slower time course to access the matrix subject suggests that comprehenders initially access the local subject position when retrieving an antecedent for *ziji*, even if that position does not contain an acceptable antecedent. The results of our simulations suggest that this misretrieval of the local subject is not merely due to a higher resting activation for local subject positions compared to more distant subject positions. Instead, the models suggest that comprehenders attempt to use retrieval cues to limit search to the local syntactic domain. This supports the key claims of the Local Search hypothesis: comprehenders attempt to limit retrieval to the local clause, even for dependencies that are not strictly clause-bounded. This suggests that in at least some cases, locality effects in processing do not simply reflect decay and interference processes. In some cases, they additionally reflect a search strategy that favors the retrieval of syntactically local dependents.

One interesting finding from Experiment 1 is the individual variation in the retrieval dynamics observed across participants. Four of the 17 participants showed substantially faster retrieval of the long-distance antecedent than the local antecedent. For these participants, the average speed advantage seen for long-distance antecedents was 343 ms. This variation raises the possibility that the positional cues used to retrieve an antecedent are under strategic control, such that these four participants were able to prioritize retrieval of the highest subject over the local subject. Additionally, two of the remaining 13 participants showed a substantial rate advantage for the local conditions, driven by extremely fast retrieval speeds for local *ziji* conditions. The extremely rapid growth of these participants' SAT functions suggests that they may have adopted a distinct strategy for determining whether *ziji* was licensed in our experiment, perhaps one based on familiarity with an animate referent rather than full retrieval of an antecedent. Although we believe it is important to understand the variation observed across our participants, we caution that these suggestions are for the moment highly speculative. Further research is necessary to determine the exact ways in which memory search strategies are subject to strategic and individual variation.

We presented a model of the Local Search strategy that relies on a direct access memory architecture. On this model, the slowdown for retrieving the matrix antecedent reflects the fact that comprehenders must execute multiple retrieval operations to recover the distant antecedent in the face of a substantive locality bias in retrieval. However, the data are also compatible with a serial scan mechanism that operates over syntactic structures. This is compatible with previous claims about the mechanisms that allow the recovery of order or positional information in retrieval (McElree and Dosher, 1993; Gronlund et al., 1997). On this model, the present results do not reflect misretrieval of the local subject, but rather a backwards process of traversing the parse until an acceptable antecedent is encountered. Although existing SAT data provide evidence against the use of serial search processes for a number of linguistic dependencies, it is possible that a serial search process is applied uniquely to syntactic binding dependencies. Indeed, Berwick and Weinberg (1984) make an argument on computational grounds for just such a serial, backwards search process for the retrieval of a bound anaphor's antecedent. However, our present results do not distinguish between these two distinct mechanisms.

Although we have argued that our simulations point to a role for a Local Search strategy in memory access, it is true that this argument rests on a number of modeling assumptions that we made. It is possible that the SAT results reflect an overwhelming activation advantage for the local subject that is not captured in our implemented retrieval model, which might lead to slowed access to the distant subject even without the use of positional cues. One way this might occur is if the local subject were available in the focus of attention at the point of processing the anaphor, thus obviating the need for any retrieval process (McElree, 2006; Jonides et al., 2008). This interpretation seems less likely in light of findings that indicate that the focus of attention is extremely limited in size and scope, possibly corresponding to just one task-relevant encoding (McElree and Dosher, 1989; McElree, 1998). If only one element occupies focal attention before *ziji* is processed, it is likely to be the verb, although it is difficult to generalize from findings about the scope of attention in recognition memory tasks to sentence processing. The data on the capacity of the focus of attention is somewhat sparser for connected linguistic representations, which have considerably richer structure than lists. However, it has been shown that opening a new clause displaces the contents of focal attention (McElree et al., 2003; Wagers and McElree, 2009), and so the adverbial clause that intervened between the subject and the verb in Experiment 1 is likely to have displaced the local subject from active memory.

A second possibility is that the local subject is reactivated at the verb that precedes *ziji*. Although our model accounted for a process of local subject reactivation prior to the anaphor, it is possible that the boost given to the local subject due to this reactivation is substantially larger than our model allows for. At present we cannot rule out this possibility, but we believe that it is unlikely on empirical grounds. In particular, data from the cross-modal lexical priming paradigm show that a subject is not strongly activated above baseline while processing its verb (Nicol and Swinney, 1989, 2003). Studies that have contrasted activation of the local subject position before and after reflexive anaphors demonstrate that reactivation of the local subject is contingent on the construction of an anaphoric dependency; processing the verb alone is not sufficient to boost activation, nor is activation observed in post-verbal positions that do not contain a reflexive anaphor (see a review in Nicol and Swinney, 2003).

Finally, it should be noted that our model assumes that all retrieval cues are equally diagnostic. Although this is a plausible assumption that is common in ACT-R modeling and elsewhere (see also Clark and Gronlund, 1996; Lewis and Vasishth, 2005), recent research into how retrieval cues are combined in sentence processing does raise the possibility that syntactic and semantic cues are not equally weighted. In particular, Van Dyke and McElree (2011) argue that syntactic cues are more highly weighted than semantic cues in comprehension, and Dillon et al. (2013) argue that cue weight may vary as a function of grammatical dependency. Further work is necessary to determine whether different cues to antecedent retrieval for *ziji* are in fact differentially weighted, and if so, how differential cue weighting would influence the conclusions of the present research.

## Conclusion

The present study examined the time-course of antecedent retrieval for the Mandarin Chinese long-distance anaphor *ziji*. It was found that *ziji* is processed more quickly with a local antecedent than with a long-distance antecedent. A computational model of the retrieval process supports the conclusion that the locality advantage observed when retrieving *ziji'*s antecedent reflects an explicit local search strategy: when retrieving an antecedent, comprehenders prioritize retrieval of items within the local clause. These results suggest that locality effects in sentence processing cannot be entirely reduced to the effects of temporal decay and interference in memory.

### Conflict of interest statement

The authors declare that the research was conducted in the absence of any commercial or financial relationships that could be construed as a potential conflict of interest.

## Footnotes

^1^The parameter settings considered were: *F* = {0.08, 0.10, 0.12}; *d* = {0.4, 0.5, 0.6}, *S* = {0.75, 1.0, 1.25}; *G* = {0.75, 1.0, 1.25}. Crossing all possible parameter settings resulted in 81 unique parameterizations of the model.

^2^Fifty milliseconds is the time necessary to execute a single production step in ACT-R.
